# Mesoscopic Effects in an Agent-Based Bargaining Model in Regular Lattices

**DOI:** 10.1371/journal.pone.0017661

**Published:** 2011-03-09

**Authors:** David J. Poza, José I. Santos, José M. Galán, Adolfo López-Paredes

**Affiliations:** 1 Social Systems Engineering Centre INSISOC, Valladolid, Spain; 2 Área de Organización de Empresas, Departamento de Ingeniería Civil, Universidad de Burgos, Burgos, Spain; University of Maribor, Slovenia

## Abstract

The effect of spatial structure has been proved very relevant in repeated games. In this work we propose an agent based model where a fixed finite population of tagged agents play iteratively the Nash demand game in a regular lattice. The model extends the multiagent bargaining model by Axtell, Epstein and Young [1] modifying the assumption of global interaction. Each agent is endowed with a memory and plays the best reply against the opponent's most frequent demand. We focus our analysis on the transient dynamics of the system, studying by computer simulation the set of states in which the system spends a considerable fraction of the time. The results show that all the possible persistent regimes in the global interaction model can also be observed in this spatial version. We also find that the mesoscopic properties of the interaction networks that the spatial distribution induces in the model have a significant impact on the diffusion of strategies, and can lead to new persistent regimes different from those found in previous research. In particular, community structure in the intratype interaction networks may cause that communities reach different persistent regimes as a consequence of the hindering diffusion effect of fluctuating agents at their borders.

## Introduction

The role that norms play as regulator mechanisms of certain aspects of social, economic and organizational behaviours has been thoroughly studied in the social sciences [2], [3]. Once a norm has been established, it acts as a self-reinforcement mechanism of behaviour. However, the emergence, diffusion and collapse of social norms are, in general, exempt from explicit mechanisms of control.

There are different kinds of norms depending on the type of social interaction. Concretely, in the economics field, an important research effort is focused on understanding the emergence of norms that determine the property distribution in a community. Thus, in contrast to the equity norm that leads to “distributive justice” and fair division of goods in some communities, there is also evidence of systematic persistence of discriminatory norms that allocate different shares of a resource according to some individual characteristic or group membership.

Evolutionary game theory is a powerful framework to analyse this type of norms formally. In particular, the Nash bargaining game [4] is often used as a simple archetypical model of economic interaction and good distribution. Succinctly, the two-player Nash bargaining game consists of two players that have to divide a sum of money among them. The payoff for each player is the amount of money they asked for, but if the sum of the demands exceeds the total, they both obtain nothing.

If the game is played repeatedly among an infinite population of players that are randomly paired up and change their strategy according to the replicator dynamics then, given a particular initial condition, it is possible to compute the distribution of strategies in the population over time [5]. Notwithstanding, the influence of such assumptions has proved very relevant for the results of evolutionary game models [6]. This is particularly relevant given that such assumptions are not always easy to justify.

In 2001, Axtell, Epstein and Young [1] proposed an agent-based model (henceforth AEY's model) to understand the transient and the asymptotic dynamics of the Nash demand game in a finite population. They simplified the analysis considering just three possible demands: low (L), medium (M) and high (H). They proved that different self-reinforcing norms can emerge spontaneously. These emergent norms may be completely different from one another even though all the agents of the population have exactly the same behavioural rule. Which particular norm appears first depends on initial conditions and on purely accidental events, such as the specific pair of agents that happened to be (randomly) paired at a certain time.

To obtain these conclusions Axtell et al. conduct their analysis in two parts. Initially they study the dynamics of a population of indistinguishable agents with the capacity to store in their memories the strategies played by their opponents in the last encounters. Each agent uses this information to form an expectation about her opponent's strategy, assuming that the probability of the next demand equals the relative frequency of the remembered experiences in the last encounters. Given that belief, each agent responds with a “noisy best reply”, i.e. a best reply with a small probability of selecting a random demand. One of the norms that can emerge in this setting is the so-called “equity norm”, i.e. a self-fulfilling situation where everyone expects the others to demand M and, as a consequence, everyone demands M; this behaviour, in turn, confirms the expectations that everyone already has, thus closing a self-consistent loop. Axtell et al. point out that the “equity norm” is the unique stochastically stable state of the game (see Young [7], [8] for a comprehensive analysis of the required conditions to obtain this conclusion), but they also find other persistent stable fractious states, in which players play repeatedly L or H, but never M.

More interestingly, in the second part of their analysis, Axtell et al. endow each individual agent with one of two possible tags (which can be recognised by all, but has no initial meaning) and with the ability to remember both the past behaviour of her opponents and their tag. In this second setting, they find that a new stable state can endogenously emerge, in addition to the ones previously observed. In this new state, agents behave differently within and outside their own tag group, so the state was naturally labelled “segregation”. The implications of Axtell et al.'s finding are astonishing: a discriminatory norm in which property is unequally distributed based on observable characteristics that are initially meaningless, may not only emerge but even perpetuate for long, as a consequence of the self-reinforcing nature of the dynamics. These results are very suggestive from a social point of view when we associate the concept of tag in the model with some social or cultural trait such as race, gender or age, which may condition people's behaviour in human societies. Using the model as reference, the emergence of a rich variety of collective outcomes can be explained. An example would be the situation where a divided underclass is oppressed by a unified elite: this would correspond in the model to a state where the elite group systematically plays H against the oppressed group (who responds optimally playing L) and plays equitable (M) among themselves, while the discriminated group is stuck in a fractious state. The replicator dynamics embeds two important assumptions: infinite populations (which is the hypothesis relaxed by Axtell et al. [1]) and random pairings. The assumption that pairings are random can be understood as an abstraction of persistent bargaining interaction with strangers. However, in some contexts this may be unrealistic; agents may interact only with just a small number of other agents with which they are in direct contact [9], [10]. In those cases the global interaction assumption can be removed and we can analyze the effect of a given social or spatial structure.

Introducing structure in the population implies that the probability of interaction between two agents depends on the specific pair of agents. The structure of the population can be usefully represented by means of a graph or network that describes the interaction connectivity. Ohtsuki et al. [11], [12] argue that in a general case, the structure should be described by two graphs, one representing the interaction of the game played and a second one representing the interaction of the adoption or learning mechanism. Usually both graphs are considered the same. The effect of many different types of graphs in games has been investigated, examples of which include the analysis of iterated 2×2 games such as the Prisoner's Dilemma on regular lattices [13]–[15], Erdos-Renyi [15]–[17], small-world [15], [18], scale-free [16], [17], [19]–[23] or real networks [24], [25], the analysis of the snowdrift game on lattices [26], [27], small-world [28], [29] or scale-free [19]–[21] networks, and n-person games such as public good games on lattices [30]–[33] or the minority game on small world networks [34], [35] (some reviews can be found at [6], [36], [37]). In this article we have extended the analysis of norm diffusion in a population considering AEY's model as a framework. We have studied the influence of the topology on the results of the game. To this aim, we have considered the spatial dimension of the game by introducing a regular spatial structure. We have also modified the original model by adding a new behavioural rule that requires less cognitive abilities than those required in the original paper. When agents use this behavioural rule, the segregation norm emerges more frequently, and a richer space of solutions is observed.

This work is organized as follows: first, we briefly explain the extensions and modifications that we have performed on AEY's original model. Next, we describe the results that we have obtained when agents are randomly assigned a tag. At the end of this section we discuss some cases where several persistent regimes can simultaneously emerge, and their relation with some mesoscopic topological properties. We then finish with the conclusions of this work.

## Methods

### Agent-based Model of Bargaining in a Regular Lattice

In this section we describe an agent-based model of bargaining in regular lattices based on the original tag model proposed by Axtell et al. [1]. Our model introduces a spatial restriction in the structure of interactions: agents are embedded on a regular lattice and they can only bargain with their spatial neighbours. In each time period of the model, each agent selects one of her neighbours at random to play the Nash demand game. When playing the game, each agent considers three possible demands of a pie (which is a metaphor of something that is going to be shared between two persons), i.e. *low (L)* or 30%, *medium (M)* or 50% and *high (H)* or 70%. The agents get the chosen demand if the sum of their demands does not exceed 100 percent of the pie; otherwise they both get nothing (see the payoff matrix in the Table 1). The Nash demand game represented in Table 1 has exactly three pure-strategy Nash equilibria, corresponding to the pairs (L, H), (M, M), and (H, L). These are called the equilibria of the one-shot bargaining game.

**Table 1:** Payoff matrix of the Nash demand game.

	H	M	L
H	(0,0)	(0,0)	**(70,30)**
M	(0,0)	**(50,50)**	(50,30)
L	**(30,70)**	(30,50)	(30,30)

As in Axtell et al. [1], the population of agents is divided into two groups of equal size whose members share a recognizable characteristic which has no a priori social meaning, i.e. a tag. These tags are used by the agents to select their demand in the one-shot game. To be precise, the agents' decision rule, which is identical for all individuals, is based on the agents' capacity to remember their opponent's demand in the *m* most recent interactions with opponents with the same tag (i.e. intratype interaction) and the *m* most recent interactions with opponents with the other tag (i.e. intertype interaction). These experiences constitute the agent's intratype and intertype memories. In the AEY model, an individual chooses the best reply that maximizes the expected demand considering their past experiences with similar opponents, i.e. those with the same tag. In contrast, in our model we consider a simpler decision rule, henceforth the mode rule, which dictates that individuals choose the best reply against the most frequent demand with similar opponents (ties are resolved randomly without any bias). The mode rule is cognitively less demanding than AEY's and, naturally, it induces different results than those obtained with the original rule [38].

The stochastic version of the game considers that agents may make mistakes in their decisions (or simply experiment from time to time). Hence, with probability (1−*ε*) an individual chooses the best reply and with probability *ε* she chooses one of the three possible demands at random (*low*, *medium* or *high* with the same probability).

The model has been implemented in Netlogo (http://ccl.northwestern.edu/netlogo/) and can be downloaded at this url: http://ingor.ubu.es/models/aeygrid).

**Figure 1:**
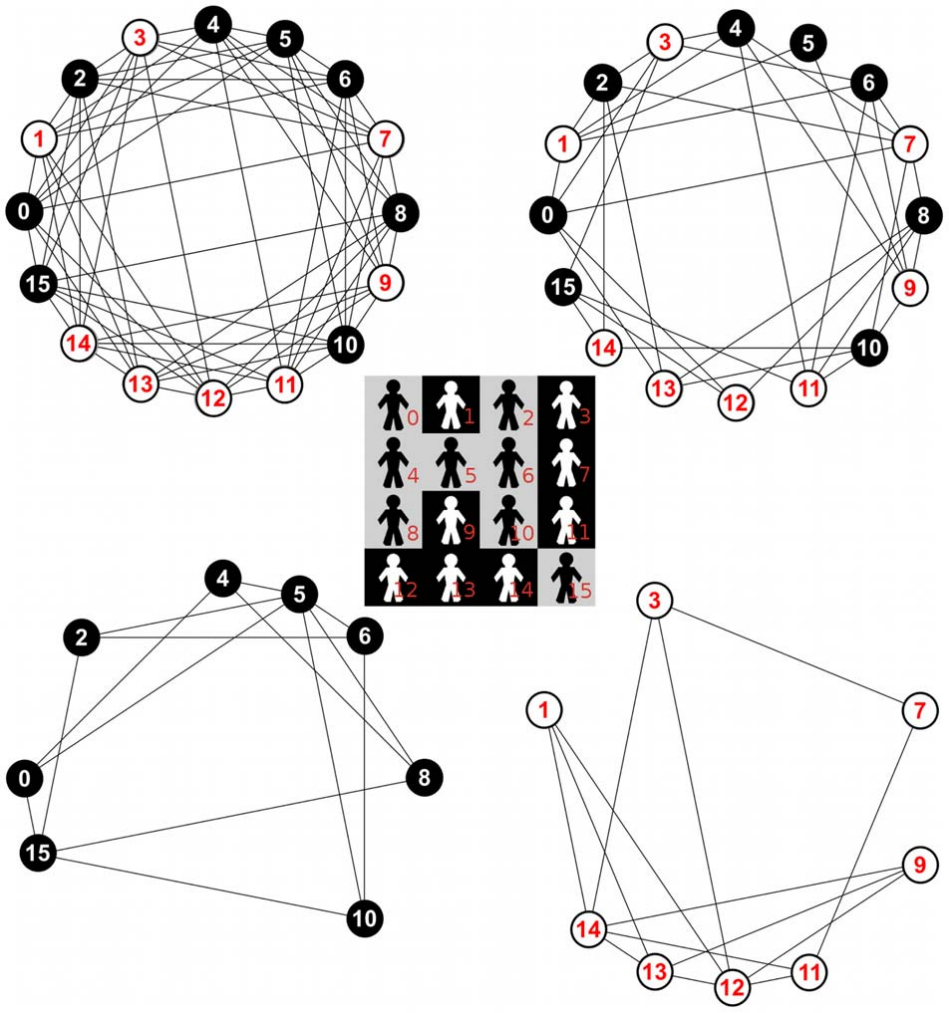
Example of a spatial distribution and its corresponding interaction networks. The spatial distribution, depicted in the centre, consists of 16 agents randomly distributed in a regular 2-dimensional toroidal lattice of 4×4 cells. The corresponding set of interaction networks are shown in the corners: (i) the complete interaction network (upper left corner), (ii) the intertype interaction network (upper right corner), (iii) the “black tag” intratype interaction network (bottom left corner), and (iv) the “white tag” intratype interaction network (bottom right corner).

We can summarize the model as follows: there is a population of *N* agents randomly distributed in a regular 2-dimensional toroidal lattice of *LxL = N* cells, each one inhabited by one and only one of the *N* agents. The population is divided exactly into two groups whose members have a distinctive tag. The number of agents is chosen satisfying simultaneously two conditions: (1) it is even, so the population can be divided exactly into two groups, (2) and its square root is an integer, so the regular lattice is square too. Each agent is endowed with two memories of length *m* to keep the demands of the two classes of tags. Memories are initialized at random. In each time period *t*, each agent randomly selects one of her 8 neighbours (radius-1 Moore neighbourhood) to play the game. The agent observes her opponent's tag and decides the best reply against the most frequent demand in her corresponding memory (i.e. intratype or intertype). However, with a small probability *ε* an agent decides randomly between the three possible demands. Afterwards both agents update their memories. Figure 1 shows the different interaction networks of a particular spatial distribution of tagged agents in a 4×4 lattice.

## Results

### Understanding the dynamics of the model

Before our computational exploration of the model, we have conducted a brief analysis within the framework of Markov Chains [39] to gain some insights about the expected dynamics and behaviour of the model. Fortunately, some aspects of this formal analysis have already been carried out for the AEY model by Axtell et al [1], and for the evolutionary model of bargaining by Young [7], [8]. In terms of markovian properties, our model shares many characteristics with these models. To represent the model as a time-homogeneous Markov Chain (THMC), we define the state of the system in a time period t as a N-dimensional vector X_t_ = {X^1^_t_, X^2^_t_,…, X^N^_t_} of 2m-tuples X^i^_t_, each one corresponding to agent i's memory of both intratype and intertype encounters (it is not necessary to use all m values of the agents' memory to represent the state space, since knowing only the memory length and two of the frequencies of each possible demand {L,M,H} is enough). Note that the spatial distribution of tags conditions the chances of intratype/intertype encounters in each period, but the possible changes that may occur in each interaction are only dependent on the particular form of the two m-tuple memories that are involved in the interaction.

The characteristics of the system dynamics are strongly determined by the presence or absence of errors (mutations in evolutionary terminology) in agents' decisions. In the absence of decision errors, i.e. the *unperturbed model*, the system has absorbing states in which sooner or later it will be trapped (if we run the model for long enough). These absorbing states are directly related with the three pure-strategy Nash equilibria of the Nash demand game, giving rise to the *equity norm* (EQ) and the *inequity norm* (IQ). The former happens when everyone in the population expects the others will demand M, and consequently everyone demands M, so the system ends reaching an absorbing state for both intratype and intertype bargaining processes, which is equitable because all agents get equal payoffs, and is also efficient (in Pareto sense) because no agent can be made better off without making another agent worse off. Apart from this, in the AEY model without spatial restrictions [1], there are also IQ absorbing states for the intertype bargaining game. An IQ equilibrium corresponds to a state in which tagged agents coordinate in one of the two asymmetric pure-strategy Nash equilibria. Whenever agents of one tag expect the others will demand L and hence they will demand H, and simultaneously the others will expect and demand the complementary decisions, the system reaches an absorbing state, which in this case is efficient but not equitable in the proportions obtained by each agent.

**Figure 2:**
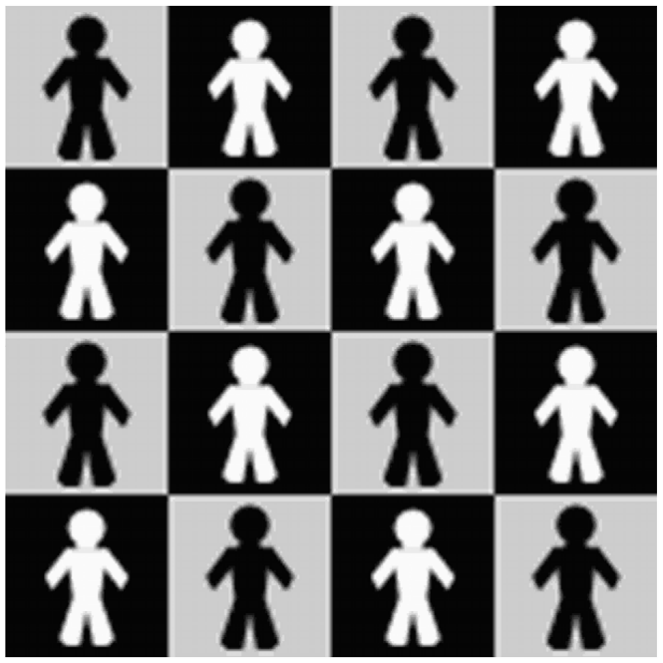
Toroidal grid of 4×4 cells with 8 white and 8 black agents distributed in the way shown. In this particular spatial distribution the system may reach the IQ state in both intratype games whenever similar tagged agents placed in the same column demand exactly the complementary quantity (L or H) of their neighbours of columns just next to them. This result is true if the intratype network is bipartite, i.e. there are no odd-length cycles.

Interestingly, additional absorbing states show up as a consequence of the imposed spatial structure. For example, a spatial distribution of 4×4 tagged agents like the one depicted in Figure 2 allows an IQ absorbing state in both intratype bargaining games, i.e black-black and white-white.

When randomness is introduced in agents' decisions (motivated by the possibility of mistakes or by a simple desire for exploration), the system becomes ergodic. In this case, there is a unique limiting distribution over the state space which determines the probability of finding the system in each of its states in the long run (e.g. lim_t→∞_P(X_t_ = i)). Such probabilities are strictly positive and independent of the initial conditions. This limiting distribution can be estimated sampling just one simulation run for a sufficiently long time, by computing the fraction of the time that the system spends in each state, i.e. the occupancy distribution [39]. In contrast to what one may expect, when the tagged model for a finite population and global interaction is asymptotically analysed, this limiting distribution concentrates only on one of the two absorbing states of the unperturbed model, the EQ. The formal demonstration of this relies on the concept of stochastic stability [8]. When some small noise exists, the EQ state is stochastically stable while IQs are not. This implies that, in the long run and for sufficiently unlikely perturbations, the system tends to spend most of the time at the EQ state. Nevertheless, Axtell et al. [1] make an interesting contribution turning the attention from the asymptotic to the transient dynamics, and showing that there are other relevant states in which the system spends a considerable fraction of the time, henceforth *persistent regimes*. In the transient evolution of the global interaction model, sometimes the system is temporarily trapped in a particular regime, called *fractious regime* (FR), in which agents alternate their demands between H and L, making the emergence of the equity norm very difficult (We keep the word fractious for consistency with the original AEY's model; but it may be worth noting that other names, such as “fluctuating agents” [16], [40], have been used in the literature for essentially the same concept, i.e. agents that intermittently change their strategy). Moreover, they show that the transition time between this fractious regime FR to the stochastically stable state EQ can be enormously long and this time grows exponentially with the number of agents and their memory length -i.e in their terminology: ergodicity is broken.

**Figure 3:**
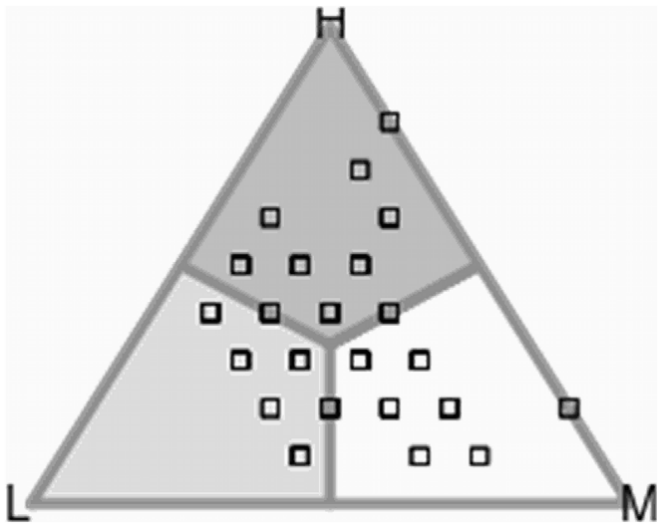
2-simplex representation of the state space used in both intratype and intertype bargaining games. The shaded regions correspond to the state subspaces in which an agent always decides one of the three possible demands {L,M,H}. For example, the light grey area at the bottom-left of the triangle represents a set of states in which the majority of the items in the memory are L, and therfore the agent will demand H. The opposite happens in the dark grey area at the top. Finally the M demand dominates the agents' memories in the white area, so the response of the agent in that area is also M. Note that with the mode-decision rule, the centre of the triangle, which is equidistant from the three vertices, corresponds to the indifferent state in which any of the three demands is equally possible.

Formally, the system is completely characterised by the vector X_t_, which can be graphically represented using a 2-simplex of the agents' states (see Figure 3). Each of the two agent i's memories keeps track of the demands made by her opponents in the m most recent intratype (or intertype) encounters, and can be represented by a vector of the relative frequencies of these demands Xi = {n_L_/m, n_M_/m, n_H_/m}, where n_L_ denotes the number of times that agent i's opponent demanded L in the m most recent intratype (or intertype) interactions. This vector corresponds in the simplex with the point 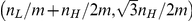. Since the memory of an agent is made by two partitions, corresponding to the past demands of the two classes of opponents, we can use two separated simplexes to represent each one.

### Most Frequent Persistent Regimes

If, following the approach in Axtell et al [1], we focus our analysis on the transient dynamics of the spatial model, the complexity of the system makes us to resort to computer simulation as methodology. We have designed a set of experiments to discover and understand the persistent regimes that emerge in the model.

The parameterization of all cases mentioned in this paper corresponds to a model of N = 100 agents randomly distributed in a regular lattice of 2-dimensional grid of 10×10 cells, each one keeping one of the N agents. Each agent is endowed with two memories of length 10 for intratype and intertype bargaining games, initialized at random. In each time period t, each agent selects one of her 8 neighbours (Moore neighbourhood) at random and decides the best reply against the most frequent demand in her memory for the type of opponent. However, with a small probability ε = 0.01 an agent decides randomly between the three possible demands {L,M,H}. Note that each time period consists of N matches, and consequently it is probable that an agent bargains more than once in each time period. We have sampled 10.000 simulation runs during T_f_ = 30.000 time periods.

The system state at the end of the simulation time can be summarized as a 3-tuple of the regimes reached by the intertype and the two intratype bargaining games {Intra-white_regime_, Intra-black_regime_, Inter_regime_}. Taking into account the characterization of the types of stable and persistent regimes described in the previous section, we may expect that if we let the system run for long enough, it will reach one of the 3^3^ possible combinations, i.e. {EQ,EQ,EQ}, {EQ,EQ,FR}, …. We define a set of simple conditions, henceforth *C1 stop conditions*, for reaching each of the persistent regimes according to their nature: the EQ state is considered reached whenever all agents in the corresponding bargaining process have at least *(1−ε)*×*m* instances of M in their memories (note that the memory vector has a finite number of instances, so we approximate (1−ε)×m to the lower integer and ε×m to the higher integer), the IQ and the FR regime are considered reached whenever all agents have at most *ε*×*m* instances of M and, moreover, in the case of the IQ state a group of agents have *(1−ε)*×*m* instances of L and the rest have *(1−ε)*×*m* of H, and in the case of FR all agents have a combination of *(1−ε)*×*m* instances of both L and H. In short, a simulation run stops when either it satisfies one of the C1 stop conditions or it reaches the final time period T_f_.

**Figure 4:**
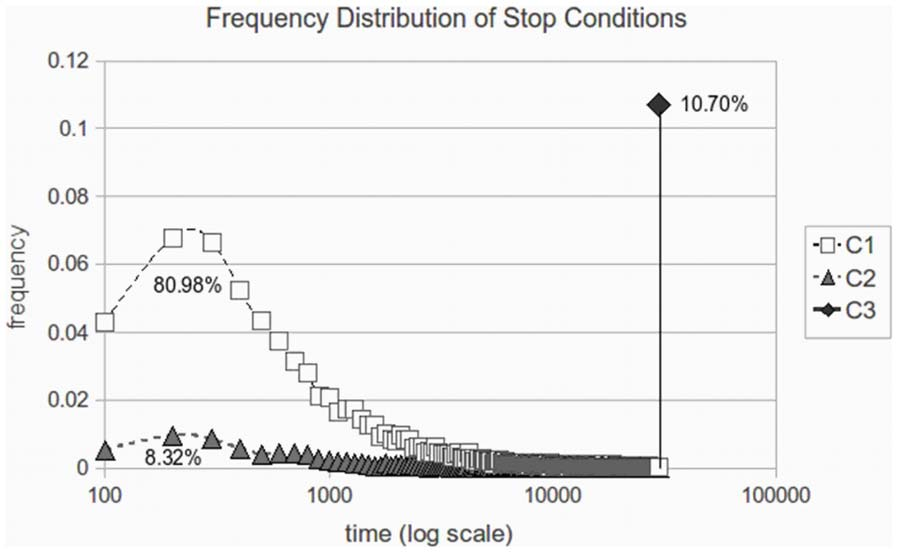
Frequency distribution of the stop conditions reached by 10.000 simulation runs. C1 represents the relative frequency of runs that reached one of the C1 stop conditions defined in section ‘Most Frequent Persistent Regimes’. C2 represents the frequency of runs that reached the C2 stop conditions defined in section ‘Isolated Bargaining Clusters’. This stop criterion extends the C1 conditions to disconnected interaction components that can randomly appear in the spatial distribution of agents on the grid. Finally C3 gathers the rest of the runs, which are analysed in section ‘Other Persistent Regimes’.

**Figure 5:**
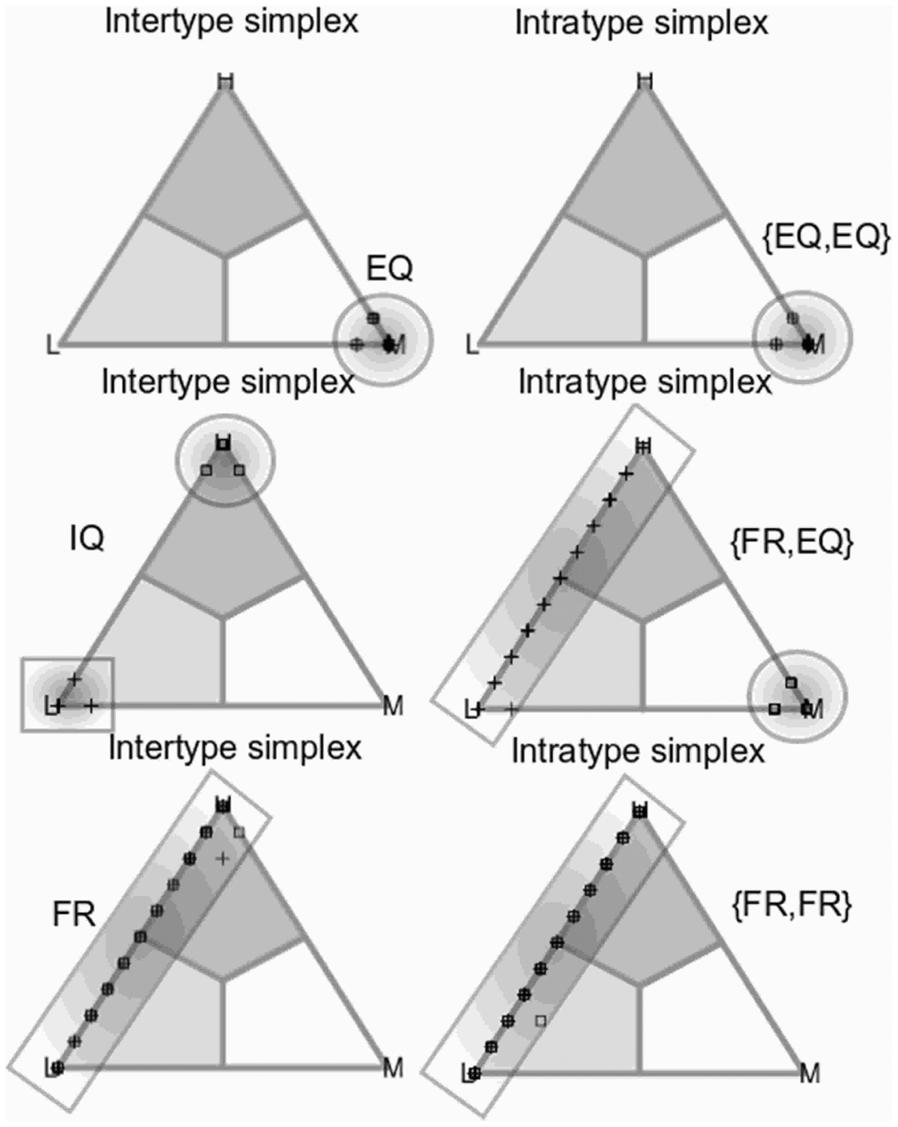
Most frequent persistent regimes of the transient dynamics for the intertype and intratype bargaining games. The bargaining between different groups (intertype) can reach the EQ state (top-left simplex), the IQ state (middle-left simplex) or the FR regime (bottom-left simplex). In the bargaining within groups (intratype) we have shown the combination of {EQ,EQ} when both groups coordinate in the EQ state (top-right simplex), {EQ,FR} when one group is in the EQ state but the other is in the FR regime (middle-right simplex), and when both groups stay in the FR regime (bottom-right simplex).

Figure 4 plots the frequency distribution of the stop conditions reached by all simulations we run. As one may expect, the system reaches one of the persistent regimes previously defined in the majority of the cases (80.98% of the runs). A relevant result is that even when the assumption of regular spatial structure of interaction, all the persistent regimes obtained in the global interaction case are also reached. Figure 5 illustrates graphically the most frequent states and regimes through a set of simplexes of some representative runs. The regime is characterized by the corresponding pair of simplexes of both intertype and intratype bargaining. Some of these states can be interpreted from a social perspective as a divided underclass oppressed by a unified elite, as class distinctions, discriminatory regimes, etc. (see [1] for a deeper insight on some interpretations).

### Isolated Bargaining Clusters

Although the analysis of the simulation results described in the previous section explains more than 80 percent, it still leaves out a significant set of them. A preliminary visual exploration of some anomalous cases gives us a possible answer: the presence of disconnected groups of agents which play the bargaining game isolated from other groups. In the initialization of the model, agents are randomly distributed and consequently most of spatial distribution samples have agents of both tags dispersed in the lattice, but close enough to make the dynamics interdependent. However, sometimes this randomness produces the formation of two or more isolated groups, i.e. groups of agents who decide their (intertype or intratype) demands without any direct or indirect influence from the agents that belong to other groups. This possibility had not been considered when we defined the C1 stop conditions, so when this event happens the simulation may reach the final time period if groups evolve to different regimes. It is important to notice that the intratype and intertype interaction networks are formed in the random initialization process and are fixed until a stop criterion is reached. Other relevant research in coevolving games does not assume fixed interaction networks but instead the structure dynamically emerges as a consequence of the game. Some of these coevolutionary rules have been used to model mechanisms of learning [41], conditional dissociation [42], unilateral and mutual choice in group dynamics [43], [44], reputation-based partner choice [45] or the formation and deletion of strategy-independent links [46]–[48].

**Figure 6:**
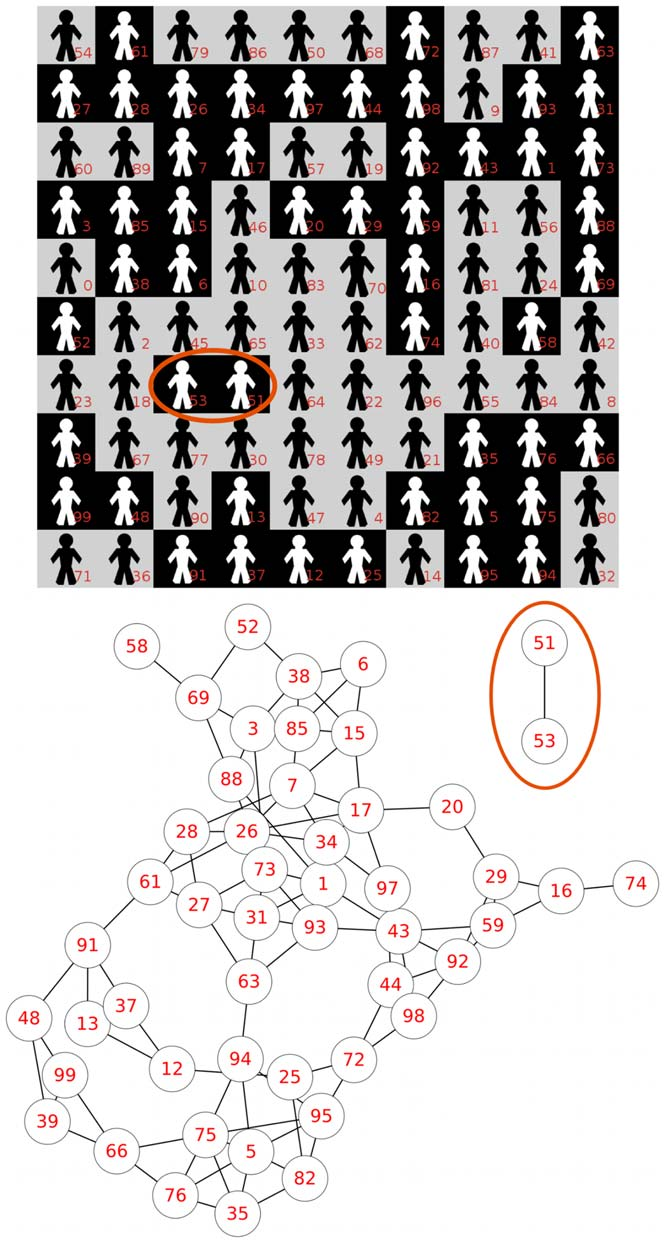
An example case that shows two disconnected groups in the white tag intratype interaction network. Upper figure: spatial distribution of agents with two disconnected groups within white-tagged agents. Lower figure: the corresponding white tag intratype interaction network in which the partition of the network is easily seen (the 51–53 couple vs the rest of white agents). Although it has not been mentioned, note that it is not difficult to identify two other disconnected groups within black agents in this example (the 60–89 couple vs the rest of the black agents).

We illustrate these cases with one of the runs that exhibits this type of spatial distribution (see Figure 6). In particular, the example run has two disconnected white-tagged groups that reach different final regimes. In order to discriminate this sort of cases we define the *C2 stop conditions* which are exactly the same conditions as C1 but applied at the level of disconnected groups -or components, in the terminology of network theory that we will use in the next section-, instead of at the level of the whole population, as we do to define C1 conditions.

Introducing the C2 stop conditions, the number of runs that end in some of the persistent regimes described so far increases until it reaches more than 89 percent of them (see Figure 4). The remaining set of runs, which end without reaching any of the expected regimes, i.e. C3 stop conditions, are analysed in detail in the next section.

Introducing the C2 stop conditions, the number of runs that end in some of the persistent regimes described so far increases until it reaches more than 89 percent of them (see Figure 4). The remaining set of runs, which end without reaching any of the expected regimes, i.e. C3 stop conditions, are analysed in detail in the next section.

### Other Persistent Regimes

The results above do not capture all persistent regimes in the game. As evidence of intensive simulation in the spatial game with random configurations, we find that there are still situations that need a much longer time to stop with one of the two criteria (C1 and C2). This fact could suggest the appearance of some other basins of attraction beyond the original AEY regimes that we have found in section ‘Most Frequent Persistent Regimes’ and the mentioned combinations of isolated states of section ‘Isolated Bargaining Clusters’. This implies that there are additional situations where the transient dynamics of the system differs from the long-run behaviour of the system.

A visual inspection of the tag spatial distribution of these cases puts forward some effects of the topology of interaction that could explain additional regimes. This happens when there are connected clusters of agents with the same tag who play different types of intratype coordination in each of the clusters. The key difference with the cases analysed in the previous section is that such clusters are indeed connected.

It seems clear that the structure of interaction has an influence on the game dynamics. We can consider the structure of intratype interaction as an undirected network where each player represents a node and there is a link between two nodes if both players can play the intratype game (i.e. they are spatial neighbours and they have the same tag). Our hypothesis is that the behaviour of the system is affected by the topological properties in the mesoscale, between the individual and the whole population, of this underlying interaction network.

One of the most relevant mesoscopic characteristics in a network is the property of community structure. Informally, a community in a network consists of a subset of nodes that are relatively densely connected to each other but sparsely connected to other dense groups [49]. This type of local structure can be easily identified in a variety of social contexts: families, friendship circles, virtual groups in the Internet, neighbourhoods, etc. In fact, there is a very rich and growing literature of networks that present community structure, going from the networks of committee and subcommittee assignments in the United States House of Representatives [50], scientific collaboration networks [51], to networks of e-mail interactions between university employees [52] or the collaboration network of jazz musicians [53]. We presume that in connected networks that present strong community structure, different communities can reach different persistent regimes, and the spread of one of the regimes to the whole connected group can be obstructed if the inter-community connectivity is low.

**Figure 7:**
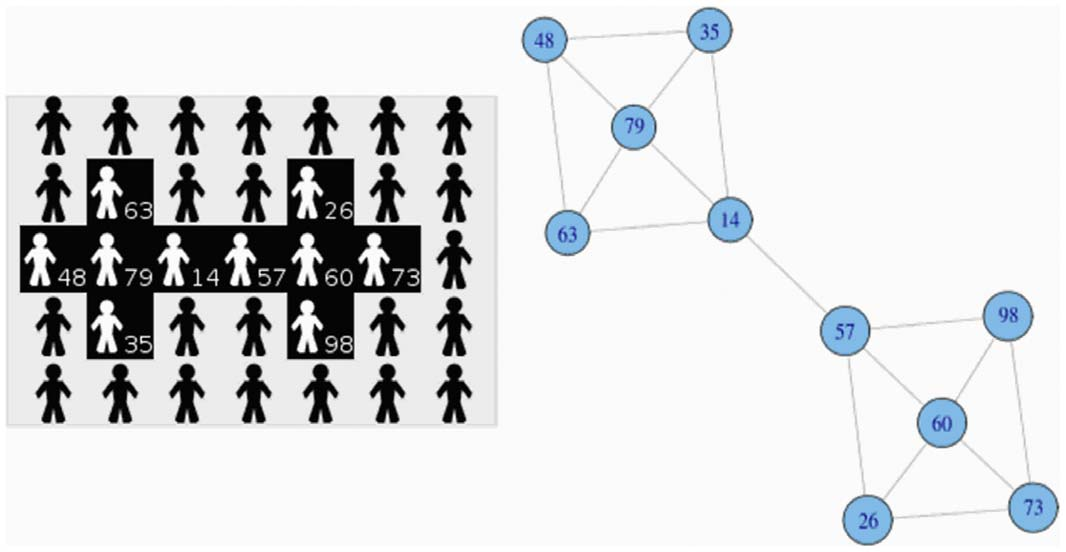
Idealized case of white-tagged players and the underlying intratype interaction network. We have analysed the effects that appear in the stylized configuration showed on the left of the figure. On the right, we represent the underlying interaction structure for the intratype game of white-tagged players.

**Figure 8:**
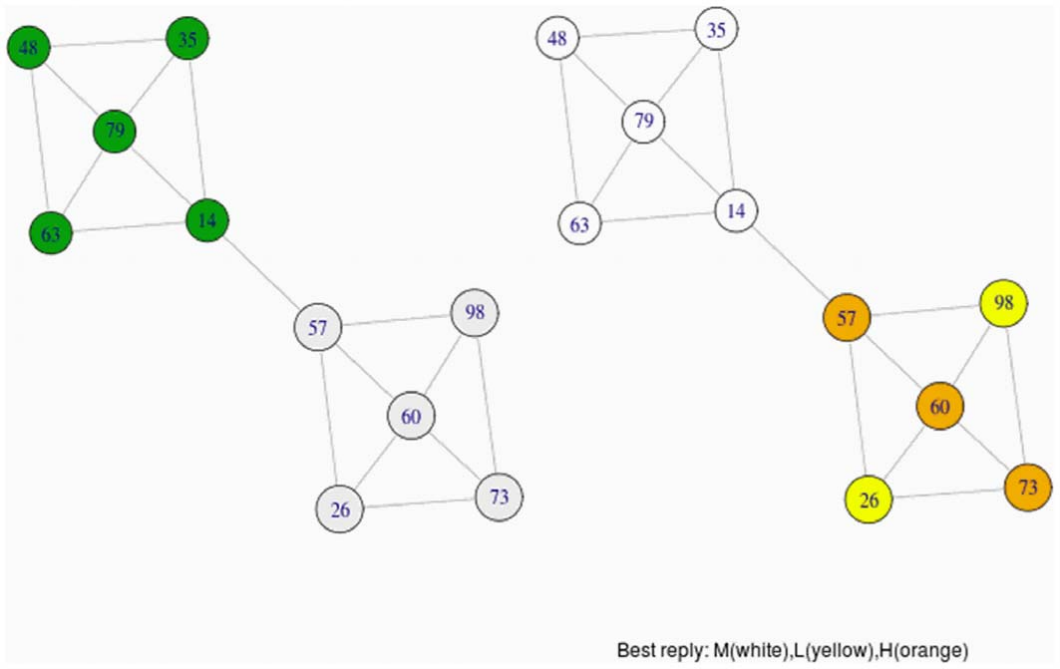
Partition in communities of the idealized case and final regimes. On the right, we can observe two different persistent regimes in the intratype game of white-tagged players depicted in Figure 7. The different regimes correspond exactly to the different communities showed on the left.

We illustrate the intuition of this phenomenon in the following idealized case. In Figure 7 we represent a certain configuration of tags and the underlying intratype interaction network of white-tagged players. Intuitively there are two communities in the network (depending on the algorithm used to identify communities, there may be other partitions in communities different to the presented in the example). If we play this game repeatedly a frequent result is showed in Figure 8. Each community reaches a different regime, stays trapped in it for a long time, and the diffusion of a general homogeneous behaviour in the game is hindered.

If we want to extend these results to more general conditions in the lattice game, we need to specify exactly how to define the concept of community beyond the intuitive and vague idea of some nodes very connected among them and sparsely linked with other communities. As a matter of fact, the problem of detecting communities is very challenging for two reasons: first, the number of possible partitions is huge for non-trivial networks, and second, but no less important, the concept (and hence the preferred definition of communities) may be domain-specific, depending on the field of application. Given this, it is not surprising that nowadays there is a wide plethora of methods based on different techniques and ideas to define and to identify communities in networks (see some recent reviews in [49], [54]–[57]).

In our analysis of the spread or lock-in of the persistent regimes, the idealized case gives us a hint to select the identifying community algorithm. We see that the edges that separate communities act as bottlenecks that enable or put obstacles to the flow of strategies. Based on this idea, Girvan and Newman [51] defined the concept of betweenness of an edge generalizing the concept of betweenness of a node by Freeman [58]. The betweenness of an edge is calculated as the number of geodesic (i.e. shortest) paths between node pairs that run through it, normalized dividing by the number of pairs of nodes. The betweenness of an edge gives us an idea of the importance of the link to stop the flow of information in the network.

The algorithm of Girvan and Newman requires calculating the betweenness of all edges in the network and removing the one with the highest betweenness, repeating the whole process until no edges remain (in case of tie, one can be randomly removed, or all can be simultaneously removed). The logic of the algorithm is based on the idea that the edges connecting communities will have comparatively high betweenness and hence, by removing them iteratively, we will separate the different components of the network that reveal the hidden community structure of the graph. The result of this algorithm is a dendrogram where horizontal cross-sections represent different possible community divisions, depending on the desired number of communities. Since the method does not provide the appropriate number of communities to split the network, the same authors [59] proposed to evaluate the divisions using the concept of modularity as the fitness function. The modularity of a partition is an index that aims to quantify how good a partition is. Partitions with high values of modularity are those in which there are dense internal connections between the nodes within clusters but only sparse connections between different clusters. Modularity compares the number of links inside a community with the expected number of links that one would find in the community if the network were randomly generated keeping the degree of every node (i.e. the number of links), but linking them randomly. Following Newman [60], the modularity Q of an unweighted and undirected network partitioned into communities can be computed as:
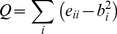
(1)where *e_ii_* denotes the fraction of all edges that have both ends in community *i*, and *b_i_* is the fraction of edges that have one or two ends in community *i*.

Given that this algorithm to partition the network formalizes the idea of information flow, we hypothesize that some additional persistent regimes can appear when each community adopts a coordinated regime except for potentially some border agents with other communities that can present a fluctuating behaviour depending on the community with which they play, and hence act as bottlenecks for the diffusion of norms between communities.

In order to check our hypothesis we have analysed the instances where simulations have not reached any of the persistent regimes considered in the previous sections: the simulations that stopped because they reached the final time period T_f_, i.e. stop condition C3. In each one of these cases we have recorded the final state of each player in the intratype game according to the definitions of section ‘Isolated Bargaining Clusters’. If the agent did not reach any of the predefined states based on her memory, we classify her as “regime not established”.

In those games, we have also exported the topology of each component of the underlying intratype interaction network and applied the Girvan-Newman algorithm maximizing the modularity to identify the different communities. We can compare the partition given by the algorithm with the final behavioural state of the players in the game. If the mesoscopic topology conditions the spread and diffusion of strategies in the lattice, the state of the players should be homogeneous in each community except for potentially some nodes that are at the border of the community. We define a node as border in a community if she has a link to another player that belongs to a different community. When two connected communities stabilize in a different regime, the agents that are at the border should present a flipping strategy, as a consequence of their exposure to different regimes. In fact, given the construction of the Girvan-Newman algorithm, the interaction of an agent that is at the border of a community with the neighbour community is done by means of links of high betweenness. In general, the frequency of interaction of those agents with players in the neighbour community is going to be lower than with players in their own community. In terms of diffusion of regimes this fact is crucial, since in order to change their strategy they would need to play very often with players from the other communities, which is against the chances imposed by the topology. Agents at the border act as buffers and stabilizers of the diffusion of regimes.

**Figure 9:**
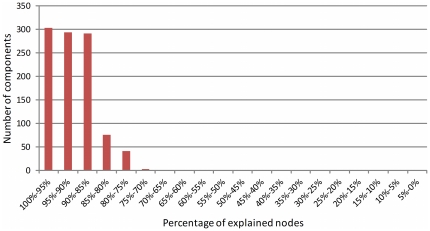
Number of components analysed that have a given percentage of explained nodes (i.e. nodes that have a homogeneous strategy with their communities, or border nodes with a different strategy to that in their community).

In the 1007 components from simulations that finished with the C3 criterion, we have computed the number of nodes that have a homogeneous strategy with the community where they belong, and the number of border players that have a different behaviour. The number of nodes in this category accounts for 91.7%. We represent in Figure 9 the percentage of nodes explained in the final state of the 1007 components.

**Figure 10:**
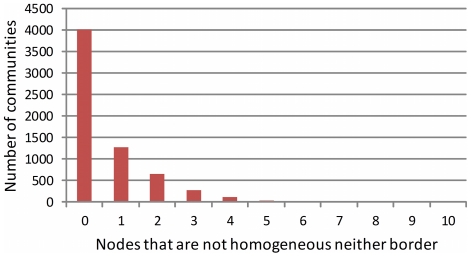
Number of communities identified by the Girvan-Newman algorithm which have none or more nodes that are not homogeneous neither border.

Our community analysis of the 1007 networks has identified 6,366 communities. We have also computed the number of players within a community proposed by the algorithm that have a discordant behaviour according to our hypothesis. Results are presented in Figure 10. As we can see more than 60% of the communities have exactly the expected behaviour.

The 8% of nodes that present a strategy discordant with the expected behaviour can be explained by different reasons. First of all, it is important to notice that the model is stochastic, and hence some randomness is going to be present. This randomness may introduce important inertia in the analysis of the state of the players. Another factor could be that we are stopping the simulations after 30 000 ticks, which for some complex topologies may be insufficient to converge to a complete persistent regime. Apart from that, we should keep in mind that the topologies analysed are obtained from complete random initialization of agents in the lattice which may produce strange topologies. The partition in communities of such topologies can be different depending on the algorithm used. The Girvan-Newman algorithm is very appealing to explain diffusion processes because it is based on a centrality measure but other algorithms are better at maximizing the modularity [54]. It is possible, therefore, that other algorithms give us other partitions that improve the explanation based on the Girvan-Newman algorithm. In any case, this study has shown the significant effect of the mesoscopic interaction structure in the spatial diffusion of strategies of the game in the lattice.

## Discussion

In this work we have addressed the effect of a regular spatial structure on the Nash bargaining game in a finite population of tagged agents. We have showed that all the transient regimes proposed by Axtell et al [1] can also be present in the lattice game. More interestingly, depending on the particular tag distribution of agents through the grid we have found some topological properties that explain the diffusion of the agent's strategies in the lattice. We have showed that isolated clusters of intra or inter type of interaction can reach different persistent regimes. Moreover, we have proved the influence of the topology in understanding new stable regimes different from those found by Axtell et al [1]. To explain their appearance and persistence, we have based our analysis on the mesoscopic properties of the interaction structure, concretely in the community structure of the network of interaction. Using the Girvan-Newman algorithm based on the edge betweenness and the concept of modularity to identify communities, we can understand the behaviour of many of the nodes of the simulation that do not reach any of the previous described regimes. Although the results can be dependent on the rules of the game, they may explain the emergence of different norms of economic interaction and resource allocation among different spatial groups, not only if the groups are isolated and do not communicate among them, but also if the interaction among groups has community structure.

The findings of these mesoscopic effects in a property distribution game strongly corroborate the relevance of the arguments previously exposed by authors like Lozano et al. [24], [25] in the evolutionary Prisoner's Dilemma, Roca et al. [61] and Tomassini and Pestelacci [62] in cooperation dilemmas, and similar phenomena also described by Castelló et al. [63], [64] in the context of dynamical models of competing options. Although the game played on the spatial substrate is different from the games explored by these authors, the mechanism that prevents the homogenization of a general strategy in the population is very similar. They use the idea of topological traps [61], [63], [64] (i.e. links between nodes of different degrees in regions with few or no redundant paths) to explain why “homogeneous strategy waves” do not propagate over the network uniformly. Since we are partitioning the network using a methodology based on the concept of betweenness, we are indirectly detecting the notion of topological traps in the borders between communities, as our results show.

## References

[1] Axtell RL, Epstein JM, Young HP, Durlauf SN, Young HP (2001). The Emergence of Classes in a Multi-Agent Bargaining Model.. Social Dynamics.

[2] Coleman JS (1990). Foundations of social theory.

[3] Kandori M (1992). Social Norms and Community Enforcement.. Review of Economic Studies.

[4] Nash JF (1950). The Bargaining Problem.. Econometrica.

[5] Skyrms B (1996). Evolution of the social contract.

[6] Roca CP, Cuesta JA, Sánchez A (2009). Evolutionary game theory: Temporal and spatial effects beyond replicator dynamics.. Physics of Life Reviews.

[7] Young HP (1998). Individual strategy and social structure. An evolutionary theory of institutions.

[8] Young HP (1993). An Evolutionary Model of Bargaining.. Journal of Economic Theory.

[9] Alexander J, Skyrms B (1999). Bargaining with Neighbors: Is Justice Contagious?. The Journal of Philosophy.

[10] Charness G, Corominas-Bosch M, Frechette GR (2007). Bargaining and network structure: An experiment.. Journal of Economic Theory.

[11] Ohtsuki H, Pacheco JM, Nowak MA (2007). Evolutionary graph theory: Breaking the symmetry between interaction and replacement.. Journal of Theoretical Biology.

[12] Ohtsuki H, Nowak MA, Pacheco JM (2007). Breaking the symmetry between interaction and replacement in evolutionary dynamics on graphs.. Physical Review Letters.

[13] Fort H, Pérez N (2005). The Fate of Spatial Dilemmas with Different Fuzzy Measures of Success.. Journal of Artificial Societies and Social Simulation.

[14] Nowak MA, May RM (1992). Evolutionary Games and Spatial Chaos.. Nature.

[15] Perc M, Wang Z (2010). Heterogeneous Aspirations Promote Cooperation in the Prisoner's Dilemma Game.. PLoS ONE.

[16] Gómez-Gardeñes J, Campillo M, Floría LM, Moreno Y (2007). Dynamical organization of cooperation in complex topologies.. Physical Review Letters.

[17] Poncela J, Gómez-Gardeñes J, Floría LM, Moreno Y (2007). Robustness of cooperation in the evolutionary prisoner's dilemma on complex networks.. New Journal of Physics.

[18] Fu F, Liu LH, Wang L (2007). Evolutionary Prisoner's Dilemma on heterogeneous Newman-Watts small-world network.. European Physical Journal B.

[19] Perc M (2009). Evolution of cooperation on scale-free networks subject to error and attack.. New Journal of Physics.

[20] Santos FC, Pacheco JM (2005). Scale-Free Networks Provide a Unifying Framework for the Emergence of Cooperation.. Physical Review Letters.

[21] Santos FC, Pacheco JM, Lenaerts T (2006). Evolutionary dynamics of social dilemmas in structured heterogeneous populations.. Proceedings of the National Academy of Sciences of the United States of America.

[22] Szolnoki A, Perc M, Danku Z (2008). Towards effective payoffs in the prisoner's dilemma game on scale-free networks.. Physica A: Statistical Mechanics and its Applications.

[23] Poncela J, Gómez-Gardeñes J, Floría LM, Moreno Y, Sánchez A (2009). Cooperative scale-free networks despite the presence of defector hubs.. Europhysics Letters.

[24] Lozano S, Arenas A, Sánchez A (2008). Community connectivity and heterogeneity: Clues and insights on cooperation on social networks.. Journal of Economic Interaction and Coordination.

[25] Lozano S, Arenas A, Sánchez A (2008). Mesoscopic structure conditions the emergence of cooperation on social networks.. PLoS ONE.

[26] Hauert C, Doebeli M (2004). Spatial structure often inhibits the evolution of cooperation in the snowdrift game.. Nature.

[27] Sysi-Aho M, Saramäki J, Kertész J, Kaski K (2005). Spatial snowdrift game with myopic agents.. European Physical Journal B.

[28] Tomassini M, Luthi L, Giacobini M (2006). Hawks and Doves on small-world networks.. Physical Review E - Statistical, Nonlinear, and Soft Matter Physics.

[29] Han-Xin Y, Kun G, Xiao-Pu H, Bing-Hong W (2008). Evolutionary snowdrift game on heterogeneous Newman Watts small-world network.. Chinese Physics B.

[30] Helbing D, Szolnoki A, Perc M, Szabo G (2010). Punish, but not too hard: How costly punishment spreads in the spatial public goods game.. New Journal of Physics.

[31] Helbing D, Szolnoki A, Perc M, Szabo G (2010). Evolutionary establishment of moral and double moral standards through spatial interactions.. PLoS Computational Biology.

[32] Helbing D, Szolnoki A, Perc M, Szabo G (2010). Defector-accelerated cooperativeness and punishment in public goods games with mutations.. Physical Review E.

[33] Szolnoki A, Perc M (2010). Reward and cooperation in the spatial public goods game.. Europhysics Letters.

[34] Chen J, Quan H (2009). Effect of imitation in evolutionary minority game on small-world networks.. Physica A: Statistical Mechanics and its Applications.

[35] Kirley M (2006). Evolutionary minority games with small-world interactions.. Physica A: Statistical Mechanics and its Applications.

[36] Szabo G, Fath G (2007). Evolutionary games on graphs.. Physics Reports.

[37] Perc M, Szolnoki A (2010). Coevolutionary games-A mini review.. BioSystems.

[38] Poza D, Villafañez F, Pajares J, Lopez-Paredes A, Hernandez C (2011). New insights on the Emergence of Classes Model.. Discrete Dynamics in Nature and Society.

[39] Izquierdo LR, Izquierdo SS, Galán JM, Santos JI (2009). Techniques to understand computer simulations: Markov chain analysis.. Journal of Artificial Societies and Social Simulation.

[40] Floría LM, Gracia-Lázaro C, Gómez-Gardeñes J, Moreno Y (2009). Social network reciprocity as a phase transition in evolutionary cooperation.. Physical Review E - Statistical, Nonlinear, and Soft Matter Physics.

[41] Skyrms B, Pemantle R (2000). A dynamic model of social network formation.. Proceedings of the National Academy of Sciences of the United States of America.

[42] Izquierdo SS, Izquierdo LR, Vega-Redondo F (2010). The option to leave: Conditional dissociation in the evolution of cooperation.. Journal of Theoretical Biology.

[43] Yamashita T, Izumi K, Kurumatani K (2005). An investigation into the use of group dynamics for solving social dilemmas.. Lecture Notes in Artificial Intelligence.

[44] Yamashita T, Axtell RL, Kurumatani K, Ohuchi A (2004). Investigation of mutual choice metanorm in group dynamics for solving social dilemmas.. Lecture Notes in Artificial Intelligence.

[45] Fu F, Hauert C, Nowak MA, Wang L (2008). Reputation-based partner choice promotes cooperation in social networks.. Physical Review E - Statistical, Nonlinear, and Soft Matter Physics.

[46] Szolnoki A, Perc M (2009). Resolving social dilemmas on evolving random networks.. Europhysics Letters.

[47] Szolnoki A, Perc M (2009). Emergence of multilevel selection in the prisoner's dilemma game on coevolving random networks.. New Journal of Physics.

[48] Wu B, Zhou D, Fu F, Luo Q, Wang L, Traulsen A (2010). Evolution of cooperation on stochastic dynamical networks.. PLoS ONE.

[49] Porter MA, Onnela JP, Mucha PJ (2009). Communities in networks.. Notices of the American Mathematical Society.

[50] Porter MA, Mucha PJ, Newman MEJ, Friend AJ (2007). Community structure in the United States House of Representatives.. Physica A: Statistical Mechanics and its Applications.

[51] Girvan M, Newman MEJ (2002). Community structure in social and biological networks.. Proceedings of the National Academy of Sciences of the United States of America.

[52] Guimerà R, Danon L, Díaz-Guilera A, Giralt F, Arenas A (2006). The real communication network behind the formal chart: Community structure in organizations.. Journal of Economic Behavior and Organization.

[53] Gleiser P, Danon L (2003). Community Structure in Jazz.. Advances in Complex Systems.

[54] Danon L, Díaz-Guilera A, Duch J, Arenas A (2005). Comparing community structure identification.. Journal of Statistical Mechanics: Theory and Experiment.

[55] Fortunato S (2010). Community detection in graphs.. Physics Reports.

[56] Newman MEJ (2004). Detecting community structure in networks.. European Physical Journal B.

[57] Lancichinetti A, Saramäku J, Kivelä M, Fortunato S (2010). Characterizing the community structure of complex networks.. PLoS ONE.

[58] Freeman LC (1977). A set of measures of centrality based upon betweenness.. Sociometry.

[59] Newman MEJ, Girvan M (2004). Finding and evaluating community structure in networks.. Physical Review E - Statistical, Nonlinear, and Soft Matter Physics.

[60] Newman MEJ (2004). Fast algorithm for detecting community structure in networks.. Physical Review E - Statistical, Nonlinear, and Soft Matter Physics.

[61] Roca CP, Lozano S, Arenas A, Sánchez A (2010). Topological traps control flow on real networks: The case of coordination failures.. PLoS ONE.

[62] Tomassini M, Pestelacci E (2010). Evolution of coordination in social networks: A numerical study.. International Journal of Modern Physics C.

[63] Castelló X, Toivonen R, Eguíluz VM, Saramäki J, Kaski K (2007). Anomalous lifetime distributions and topological traps in ordering dynamics.. Europhysics Letters.

[64] Toivonen R, Castelló X, Eguíluz VM, Saramäki J, Kaski K, San Miguel M (2009). Broad lifetime distributions for ordering dynamics in complex networks.. Physical Review E - Statistical, Nonlinear, and Soft Matter Physics.

